# Influence of implant-supported prosthesis design on peri-implant health: a cross-sectional study

**DOI:** 10.4317/medoral.27464

**Published:** 2025-08-16

**Authors:** Ahmad Alahmari, Rui Figueiredo, Marta García-García, Javier Mir-Mari, Eduard Valmaseda-Castellón, Alba Sánchez-Torres

**Affiliations:** 1 Orcid ID: 0009-0001-7191-1720. BDS, MS. PhD Student, Odontostomatology department, Faculty of Medicine and Health Sciences, University of Barcelona, Barcelona, Spain. Assistant Professor in Department of Periodontics and Community Dental Sciences, Division of Periodontics, College of Dentistry, King Khalid University, Abha, Saudi Arabia; 2 Orcid ID: 0000-0002-2122-6530. DDS, MS, PhD. Full Professor of Oral Surgery. Director of the Master of Oral Surgery and Implantology degree program. Faculty of Medicine and Health Sciences, University of Barcelona. Researcher at the IDIBELL Institute, Barcelona, Spain; 3 Orcid ID: 0000-0002-8052-1505. DDS, MS, PhD. Assistant Professor of the Dental degree and Professor of the Master of Oral Surgery and Implantology degree program. Faculty of Medicine and Health Sciences, University of Barcelona, Barcelona, Spain. Researcher of the IDIBELL Research Institute, Barcelona, Spain; 4 Orcid ID: 0000-0003-0108-531X. DDS, MS, PhD. Professor of the Master of Oral Surgery and Implantology degree program. Faculty of Medicine and Health Sciences, University of Barcelona, Barcelona, Spain; 5 Orcid ID: 0000-0001-9669-3187. Catedratic Professor of Oral Surgery. Director of the Master of Oral Surgery and Implantology degree program. Faculty of Medicine and Health Sciences, University of Barcelona. Researcher at the IDIBELL Institute, Barcelona, Spain; 6 Orcid ID: 0000-0002-5902-0810. DDS, MS, PhD. Full Professor of Oral Surgery. Director of the Master of Oral Surgery and Implantology degree program. Faculty of Medicine and Health Sciences, University of Barcelona. Researcher at the IDIBELL Institute, Barcelona, Spain

## Abstract

**Background:**

Peri-implant diseases are common complications that may lead to dental implant failure. An adequate prosthesis design is crucial to reduce the risk of complications, and to improve peri-implant health. The present study was carried out to assess the effect of prosthesis design upon the presence of peri-implant inflammation.

**Material and Methods:**

A cross-sectional study was conducted in patients with a single-unit implant-supported screw-retained crown. After removing the crowns, standardized photographs were made to assess several variables such as the length of the submucosal extension (SE) or the emergence angle (EA). Clinical signs of inflammation were also registered, and an experienced clinician probed the implants. The White (WES) and Pink Esthetic Scores (PES) were also recorded. Patients were classified into two groups according to the presence (positive bleeding on probing (BoP+)) or absence (negative bleeding on probing (BoP-)) of inflammation around the dental implant. Independent t-tests and one-way ANOVA were used to analyze the data.

**Results:**

A total of 90 implants were analyzed. Fifty-two implants (57.8%) had BoP+ while 38 (42.2%) had no signs of inflammation of the peri-implant tissues (BoP-). Long SE was significantly associated with BoP+ sites. The EA did not seem to be related to the presence of inflammation (*p*=0.642). PES/WES showed a negative correlation with buccal EA (r=-0.227; *p*=0.032).

**Conclusions:**

Long submucosal extensions in single-unit implant-supported crowns seem to be associated with peri-implant tissues inflammation (BoP+). A higher emergence angle on the buccal aspect was associated with poor esthetic outcomes.

** Key words:**Peri-implant diseases, peri-implantitis, dental implant, crowns, dental prosthesis, implant-supported.

## Introduction

Dental implants are a widely used therapeutic option for the replacement of missing teeth, due to their good long-term survival rates (1). However, biological complications can jeopardize the treatment outcomes. Peri-implant mucositis has been defined as a pathological condition of the peri-implant mucosa in the absence of bone loss and with clinical signs of inflammation including bleeding on probing (BoP), erythema, edema, and suppuration (2). On the other hand, the diagnostic criteria of peri-implantitis include the presence of BoP and/or suppuration; increased pocket probing depth (PPD); and progressive bone loss (3). These peri-implant disorders are common, and approximately one out of every 5 patients with dental implants will develop peri-implantitis (mean patient-based prevalence of 19.5%) (4). According to Heitz-Mayfield *et al*. (5), most of the risk indicators that have been linked with periodontal disease can also increase the risk of peri-implantitis. Thus, smokers with poor oral hygiene and with a previous history of periodontal disease seem to be more prone to peri-implantitis. Diabetes mellitus and the amount of peri-implant keratinized mucosa may also play an important role, even though the available scientific evidence is still scarce (6). The design of the prosthesis is also considered a key factor, since it may hamper access for proper hygiene around the implant (7). Indeed, several reports have shown that implant-supported restorations with convex profiles and emergence angles (EA) of 30º or more seem to be associated with marginal bone loss (MBL). In this regard, Strauss *et al*. (8) observed that an emergence angle >40º increases initial marginal bone loss, but only during the first year of loading. Furthermore, over-contoured implant prostheses might also predispose patients to peri-implantitis (9,10). Wide emergence profiles have likewise been linked with increased bone loss and apical displacement of the peri-implant biological width(11-13) .A recent cross-sectional study(14) found that a wide mucosal EA significantly increased the risk of inflammation, with angles exceeding 70º having an odds ratio (OR) of 33.5 (95% confidence interval (95%CI): 7.13-157.89). Nonetheless, the available information on this topic is still scarce, and additional research is required. On the other hand, most published studies in this field have used radiographic measurements of the prosthesis. This approach has some limitations, since it only analyzes data from the interproximal areas (8-10,15-18). Thus, studies with different assessment approaches like the use of photographs of the prosthesis might provide useful information for clinicians. In this regard, the aim of the present study was to assess the effect of the prosthesis design, namely of the buccal, palatal/lingual, mesial and distal submucosal extension (SE) lengths and emergence angles (EA), on the presence of peri-implant inflammation in single-unit implant-supported crowns. Furthermore, this research assessed possible correlations between EA and buccal SE and the esthetic outcome.

## Material and Methods

A cross-sectional study was conducted in patients treated consecutively at the Implant Maintenance Unit of the University of Barcelona Dental Hospital (Hospital Odontològic, Universitat de Barcelona, Barcelona, Spain), between March and September 2024. Patients with at least one single-unit implant-supported crown with a minimum clinical follow-up of one year after prosthetic loading were enrolled. All implants were placed at crestal level and were restored without the use of an intermediate abutment. Patients were classified according to the presence or absence of peri-implant tissues inflammation (BoP+ or BoP-). The exclusion criteria were clearly malpositioned implants (mesiodistal, apicocoronal and buccolingual directions), tissue level implants or implants with a polished collar, cemented crowns or crowns that could not be removed.

The study was approved by the local Ethics Committee (CEIm Hospital Odontològic, Universitat de Barcelona; Ref. 2024-012-1). The researchers followed the recommendations of the Declaration of Helsinki (19) and the STROBE guidelines for reporting cross-sectional studies (20). Before inclusion, all the participants were informed about the study and gave their informed consent.

**Figure 1:**
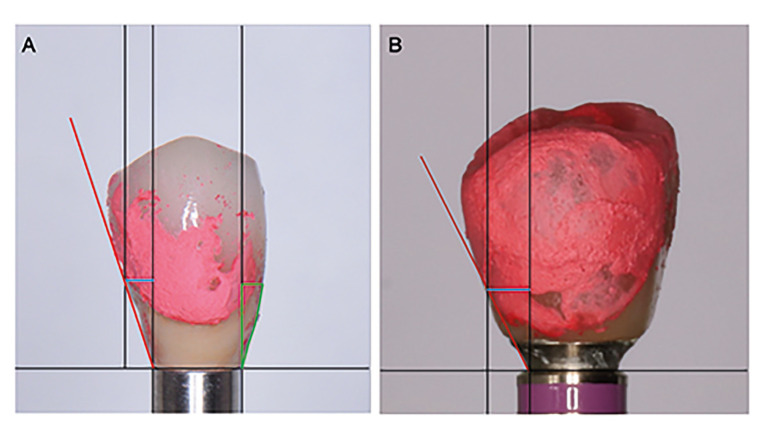
Frontal view (photograph) of the implant-prosthesis screwed into an analog and stained with occlusal spray. A) Photographic measurements of the submucosal extension (SE; blue line), emergence angle (EA; red line) and submucosal profile (SP; green area) of the prosthesis. B) Photographic variables associated with the profile of the crown. The prosthesis was considered to be over-contoured (O) if the prosthesis extension crossed the red line.

- Study sequence: A single experienced researcher (MGG) registered the following variables: age, gender, date of implant placement, smoking habit and implant position. During the same session, the researcher obtained standardized periapical radiographs using the long-cone parallel technique with a radiographic positioner, and took a frontal photograph of the implant-supported crown and peri-implant soft tissues to measure the Pink (PES) and White Esthetic Score (WES)(21). An occlusal spray (Occlu®, Hager-Werken; Duisburg, Germany) was applied to the screw-retained crown to record the mucosal margin. When the mucosal margin was not accessible (especially at the mesial and/or distal sites), a straight line was drawn from the highest point of the buccal and lingual/palatal aspects. After removing the crown, the prosthesis was placed in an implant replica fixed to a metallic cylinder. Photos were taken with a Nikon D5100 camera (Tokyo, Japan) on a 10 cm high tripod, at a distance of 15 cm. This allowed standardized photographs of the buccal, palatal/lingual, mesial and distal surfaces of all implant-supported crowns to be taken (Fig. 1).

- Clinical measurements: After prosthesis removal, a single researcher gently probed the selected single-tooth implant using a manual PCP15 periodontal probe (Hu-Friedy Inc.; Chicago, IL, USA) and registered the following variables from 6 sites per implant (mesiobuccal, buccal, distobuccal, mesiolingual, lingual and distolingual): peri-implant pocket probing depth (PPD) (distance from the mucosal margin to the base of the peri-implant sulcus/pocket); keratinized mucosa (KM) (distance from the mucosal margin to the mucogingival junction at the mesiobuccal site of each implant); biofilm (presence or absence of biofilm (4 sites per implant)); and bleeding on probing (BoP) (presence or absence of bleeding after gentle probing). This last variable was used to classify the patients into two groups: implants with (positive bleeding on probing (BOP+)) or without (negative bleeding on probing (BOP-)) inflammation of the peri-implant soft tissues.To test intra-examiner agreement, the assessment of PPD (6 sites per implant) in 7 implants was repeated after two weeks. The intra-class correlation coefficient (ICC) was 0.93 (95% confidence interval (95%CI) 0.88-0.93) (22)

- Photographic measurements: Subsequently, another blinded researcher (AA) made the photographic measurements using ImageJTM software (National Institutes of Health, Bethesda, Maryland, MD, USA). The implant diameter was used to calibrate the photographs and radiographs. The following variables were recorded:

Submucosal extension (SE): horizontal distance from the prosthesis-implant connection to the most buccal, lingual, mesial or distal point of the prosthesis marked with the occlusal spray (Fig. 1; blue line).

Prosthesis emergence angle (EA): a line parallel to the long axis of the prosthesis was drawn from the implant-prosthesis connection. Another line was drawn tangentially from the most buccal, palatal/lingual, mesial, or distal and apical part of the prosthesis marked with the occlusal spray. The angle of intersection between these lines was measured as the emergence angle (Fig. 1; red line).

Submucosal profile (SP) (mesial and distal): classified according to the angle as straight, convex, concave, or mixed. The most unfavorable angle was selected for duplicated measurements in buccal, palatal/lingual photographs (Fig. 1; green area).

Over-contouring (O): a line parallel to the long axis of the prosthesis was drawn from the implant-prosthesis connection. Another line was drawn tangentially from the most buccal, palatal/lingual, mesial, or distal and apical part of the prosthesis marked with the occlusal spray. If the prosthesis extension crossed the line, it was considered over-contoured. The total number of points with over-contouring were added up (Fig. 1).

Pink and White Esthetic Score (PES/WES): this parameter was calculated according to the criteria of Belser *et al*. (21).

- Radiographic measurements: The same blinded researcher (AA) made the radiographic measurements using ImageJTM software (National Institutes of Health, Bethesda, Maryland, MD, USA). The implant diameter was used to calibrate the radiographs.

**Figure 2:**
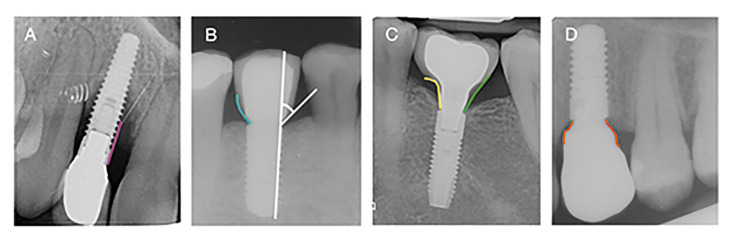
A) Bone level (BL) (pink line). B) Emergence angle (EARx) (white lines). Emergence profile (EPRx) categorized as convex (blue line). C) Emergence profile (EPRx) categorized as concave (yellow line) and straight (green line). D) Emergence profile (EPRx) categorized as mixed (orange line).

Bone level (BL): vertical distance measured mesial and distal from the most coronal part of the implant to the bone level. The most unfavorable value per implant was recorded (Fig. 2).

Radiographic emergence angle (EARx): calculated as the angle between the long axis of the implant and a line tangential to the restoration according to Katafuchi *et al*. (9) (Fig. 2).

Radiographic emergence profile (EPRx): emergence profile was categorized as straight, convex, or concave according to Katafuchi *et al*. (9). An additional category was included to account for profiles that exhibited mixed patterns (Fig. 2).

Intra-examiner agreement was measured by assessing 7 radiographs (mesial BL and mesial EARx) twice with a two-week interval. The intra-class correlation coefficient (ICC) was 0.99 (95%CI: 0.97-0.99) for BL and 0.99 (95%CI: 0.99-1.00) for EARx.

- Diagnostic variables: After collecting all the clinical and radiographic data, the patients were classified according to the diagnostic criteria of the 2017 World Workshop on the Classification of Periodontal and Peri-Implant Diseases and Conditions (23) as being healthy or presenting peri-implant mucositis, or peri-implantitis.

- Sample size calculation: The sample size was calculated using G* Power 3.0 (Heinrich-Heine-Universität, Germany). Considering an alpha error = 0.05%, a statistical power of 80% and assuming that 50% of implants with submucosal extensions > 2 mm are expected to have BoP while implants with lesser extensions would have a 30% BoP rate, a sample of 80 would be required. To compensate possible protocol deviations and dropouts, the authors decided to include a total of 90 implants.

- Statistical analysis: Data were collated and analyzed using Microsoft® Excel® (for Microsoft 365 MSO, Microsoft Corporation, Washington, DC, USA) and the SPSS version 22.0 statistical package (IBM Corp., Armonk, NY, USA). The normality of the variables was tested by means of a Shapiro-Wilk test. In the absence of a normal distribution, nonparametric tests were used for bivariate analysis, such as Fisher's exact test, the Mann-Whitney U-test or the Kruskal-Wallis test. A descriptive and bivariate analysis was performed. Independent t-tests were used to compare the differences between patient-related and prosthesis-related variables according to the presence or absence of BoP. Submucosal extension and EA were dichotomized by establishing a cut-off point for SE at 2 mm and for EA at 30º for statistical analysis. The relationship between SE, EA and biofilm with the presence of BoP was assessed using a chi-square test. Furthermore, receiver operating characteristic (ROC) curve analysis was performed to explore the cut-off points for SE and EA that predicted BoP. Finally, the correlation between SE and EA and WES and PES was analyzed using one-way ANOVA. Results with *p* ≤ 0.05 were considered statistically significant.

## Results

A total of 79 participants (36 men (45.6%) and 43 women (54.4%)) with a mean age of 52 ± 12.5 years (range 23-77) were included in the study. Ninety-two implants were screened, but two were excluded because they had intermediate abutments. Forty-four implants were placed in the maxilla and 46 in the mandible. The mean follow-up time after loading was 71 ± 29 months. Most patients in the BoP+ group (33 patients, 41.8%) did not attend maintenance appointments. On the other hand, only 11 patients (13.9%) who attended at least one peri-implant supportive therapy visit a year experienced bleeding. These differences were statistically significant (*p*=0.014). The main characteristics of the sample can be seen in Table 1.

Thirty-eight implants (42.2%) were considered healthy, 43 (47.8%) had peri-implant mucositis, and 9 (10%) presented peri-implantitis (mean marginal bone loss 3.7 ± 1 mm). No significant associations were found between the diagnosis and the prosthetic design variables. The mean PPD was 2.7 ± 1.3 mm, mean peri-implant bone loss was 1.4 ± 1.2 mm, and the mean number of bleeding points on probing was 0.3 ± 0.4. No suppuration was observed at any of the implant sites. A greater mean PPD was significantly associated with BoP+ (*p*<0.001).

Table 1 compares the implants with (BoP+; 52 implants) and without (BoP-; 38 implants) inflammation for the main implant- and patient-related variables. Submucosal extension was significantly greater in the BoP+ group versus the BoP- group on the mesial (*p*=0.046) and lingual (*p*=0.041) surfaces (Table 1).

There were no differences on comparing the BoP+ and BoP- groups with SE ≥ 2 mm and those with < 2 mm, or EA with a cut-off value of 30⁰, as shown in Table 2.

The most frequent submucosal profile on the mesial and distal aspects was a convex profile (53.9% mesial; 59.6% distal) followed by a mixed profile (24.7% mesial; 22.5% distal). There were no differences in the distribution of SP between the BoP+ and BoP- groups (*p*=0.767; *p*=0.283). Over-contouring was present in 83.3% of the implant crowns; 46.7% of the implant restorations showed over-contouring on all surfaces, with no significant differences according to BoP groups (*p*=0.814).

**Figure 3:**
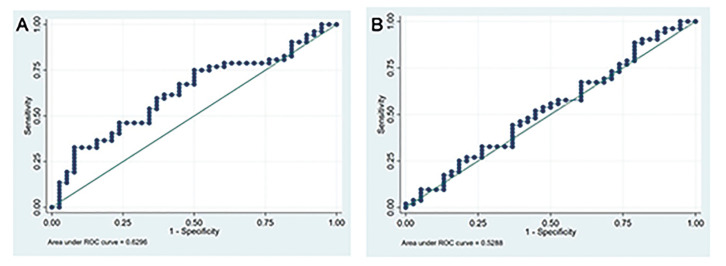
Receiver operating characteristic (ROC) curve analysis for Submucosal Extension (A) and Emergence angle (B).

The area under the SE curve for predicting BoP was 0.630 (95%CI: 0.513-0.746), and the area under the EA curve was 0.529 (95%CI: 0.406-0.652)(Fig. 3), showing that the model had low predictive power.

Regarding EPRx, the classification employed, which included an additional category (mixed profile), showed agreement with the Katafuchi classification (9), in both the mesial (*p*=0.001) and distal (*p*=0.001) measurements.

**Figure 4:**
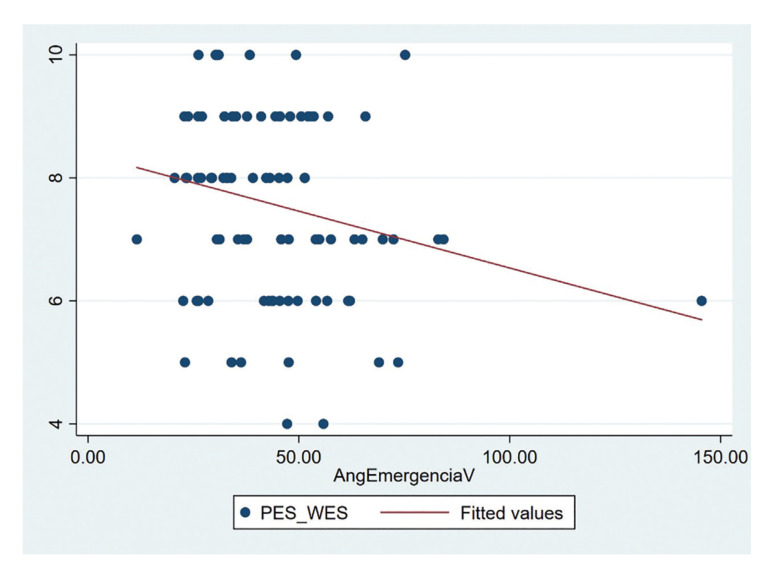
Scatter plot showing the negative correlation between the buccal emergence angle and the total value of the esthetic variable PES/WES.

Regarding the esthetic results, the mean PES and WES scores were 3.5 ± 1.4 and 4.1 ± 0.9, respectively. The mean PES and WES total score (7.6 ± 1.5) showed a negative correlation with the buccal emergence angle (r=-0.227; *p*=0.032) (Table 3) (Fig. 4).

## Discussion

The present study shows that SE seems to be related with peri-implant inflammation recorded as BoP, specifically on the mesial (*p*=0.027) and lingual (*p*=0.028) aspects. This finding may be explained by the fact that the lingual and mesial surfaces showed the highest mean extension *p-value*s in the BoP+ group (lingual: 1.6 ± 0.7 mm; mesial: 1.9 ± 1.0 mm), and this could negatively influence access for oral hygiene. On the other hand, the buccal surface showed the highest *p-value*s in both the BoP- group (2.0 ± 1.0 mm) and the BoP+ group (1.9 ± 0.9 mm), with no significant differences between them (*p*=0.877). It seems that the buccal surface, despite presenting unfavorable submucosal extensions, can be easily cleaned, thereby reducing the risk of inflammation. The present sample only included single-unit crowns, which usually have small submucosal extensions (1.7 ± 0.6 mm), and tend to allow better access for hygiene and thus less biofilm accumulation. This study also attempted to establish a critical value or cut-off point for SE that could increase the likelihood of developing peri-implant inflammation or disease. However, the receiver operating characteristic curve analysis failed to identify this cut-off point, probably because of the limited sample size and the low SE values (generally < 2 mm). Other studied variables, such as the implant-abutment connection type, did not influence the presence of bleeding.

Previous studies involving periapical radiographs found an emergence angle > 30º to be related to a greater prevalence of peri-implantitis (9,10), greater bone loss (16,18,24), or with greater biofilm accumulation and bleeding (24). In contrast, another recent study concluded that the emergence angle does not influence bone loss (17). A prospective cohort study found that an emergence angle > 40º contributed to initial marginal bone loss during the first year but had no effect on peri-implant health after 5 years (11). All these studies based their findings on radiological measurements and therefore only provide information on the distal and mesial aspects of the prosthesis. Rungtanakiat *et al*. (14) performed three-dimensional assessments using cone-beam computed tomography (CBCT) and intraoral scans, and found that other variables such as the submucosal emergence angle seem to influence the health of peri-implant tissues (24). In our case, the emergence angles of the four surfaces of the prosthesis were measured using photographs. The mesial and distal measurements concurred with the radiographic assessment, using the same methodology as Katafuchi *et al*. (9). However, the emergence angle did not seem to influence the presence of bleeding on probing. Interestingly, the highest emergence angle was found in the buccal region, with a mean of 44.1 ± 18.6º. In this regard, the horizontal bone defect caused by the bone remodeling process after tooth extraction could generate a flatter buccal extension, a greater emergence angle, more biofilm accumulation and soft tissue inflammation(24). Our findings did not support this relationship, however.

In this study, the PES and WES indexes (21) showed a negative correlation with the buccal emergence angle, i.e., the greater the buccal emergence angle, the poorer the esthetics. It seems that the design of restorations with a wide buccal emergence angle to compensate for bone defects or implant malpositioning negatively influences the esthetics of the prosthesis.

Katatuchi *et al*. (9) classified radiographic emergence profiles into straight, concave and convex. Han *et al*. (16) observed that convex profiles presented greater bone loss compared to straight or concave profiles. In the present study, a modified Katafuchi classification (9) was used, since a new category corresponding to mixed profiles was added. According to our outcomes, the prosthesis profile design (convex, concave or mixed) does not seem to influence peri-implant inflammation. Additionally, submucosal over-contouring was found in 83.3% of the cases, but this was not associated with the presence of bleeding (*p*=0.814). Therefore, we can assume that in cases of single prostheses, even in unfavorable scenarios with over-contouring, the design of the prosthesis does not seem to increase the likelihood of inflammation.

Interestingly, patients that attended peri-implant supportive therapy appointments were significantly more likely to present peri-implant inflammation. These results are consistent with previous publications by our team (25) and with the results of a recent systematic review (26).

The limited sample size constitutes one of the limitations of this study. Indeed, only 9 patients had a diagnosis of peri-implantitis. However, we focused on determining which prosthodontic factors were associated with peri-implant inflammation (BoP+). Future research should use similar methodology with a larger sample in order to determine whether the size of the submucosal extension influences bone loss. Another drawback is related with the cross-sectional nature of the study, since some variables were collected retrospectively. Also, this design limits the ability to establish cause-effect relationships. On the other hand, the present study was based on standardized measurements and used a novel methodology (photographs of supramucosally-stained restorations placed in an implant analog) that allowed the assessment of all surfaces of the restoration. Finally, the present outcomes might not be applicable to implant-supported bridges or full-arch restorations.

Future research should seek to perform a three-dimensional analysis of the submucosal area of the implant-supported crown. For this purpose, the utilization of standard tessellation language (STL) files obtained with an intraoral scanner might be especially useful.

## Conclusions

Long submucosal extensions in single-unit implant-supported crowns seem to be associated with peri-implant tissue inflammation. The emergence angle does not seem to be related with the presence of bleeding on probing but is associated with the esthetic outcome.

## Figures and Tables

**Table 1:** Patient- and implant-dependent variables.

Variables			BoP-	BoP+	Total	p-value
Patient-dependent variables	Gender (n (%))	Male	15 (19)	21 (26.6)	36 (45.6)	0.666
		Female	20 (25.3)	23 (29.1)	43 (54.4)	
	Age (years)(mean; SD)		51.7 (14.4)	52.2 (11.0)	52.0 (12.5)	0.853
	Smokers (n (%))	Yes	3 (3.8)	9 (11.4)	12 (15.2)	0.301
		No	31 (39.2)	33 (41.8)	64 (81)	
		Ex-smoker	1 (1.3)	2 (2.5)	3 (3.8)	
	Number of supportive therapy appointments in last year (n (%))	0	17 (21.5)	33 (41.8)	50 (63.3)	0.014
		≥1	18 (22.8)	11 (13.9)	29 (36.7)	
Implant-dependent variables	Position; n (%)	Incisor-canine	0	4 (4.4)	4 (4.4)	0.004†
		Premolar	13 (14.4)	16 (17.9)	29 (3.2)	
		Molar	39 (43.3)	18 (20)	57 (63.3)	
	Implant connection (n (%))	EH	18 (20)	14 (15.6)	32 (35.6)	0.827
		IC	34 (37.8)	24 (26.6)	58 (64.4)	
	Platform switching (n (%))	Yes	17 (18.9)	15 (16.6)	32 (35.6)	0.507
		No	35 (38.9)	23 (25.6)	58 (64.4)	
	Biofilm	Yes	3 (3.3)	9 (100)	12 (13.3)	0.194
		No	35 (38.9)	43 (47.8)	78 (86.7)	
	PPD (Mean; SD)		2.1 (1.2)	3.1 (1.3)	2.7 (1.3)	<0.001
	BL (Mean; SD)		1.2 (1.1)	1.5 (1.3)	1.4 (1.2)	0.233
	KM (n (%))	<2mm	10 (11.1)	11 (12.2)	21 (23.3)	0.567
		≥2mm	28 (31.1)	41 (45.6)	69 (76.7)	
	SE (mean; SD)	Buccal	2.0 (1.0)	1.9 (0.9)	2.0 (1.0)	0.909
		Lingual	1.3 (0.6)	1.6 (0.7)	1.5 (0.7)	0.041
		Mesial	1.5 (0.8)	1.9 (1.0)	1.7 (1.0)	0.046
		Distal	1.4 (1.1)	1.7 (1.4)	1.6 (0.8)	0.086
		Total	1.5 (0.6)	1.8 (0.6)	1.7 (0.6)	0.051
	EA (degrees) (Mean; SD)	Buccal	46.0 (16.7)	43.0 (20.0)	44.1 (18.6)	0.225
		Lingual	30.3 (14.2)	35.2 (15.3)	33.1 (14.9)	0.158
		Mesial	30.2 (11.6)	32.3 (11.9)	31.5 (11.8)	0.423
		Distal	27.7 (13.0)	28.5 (12.3)	28.2 (12.5)	0.800
		Total	33.5 (10.6)	34.7 (11.3)	34.2 (11)	0.642
	SP Mesial (n (%))	Straight	1 (1.1)	4 (4.5)	5 (5.6)	0.767
		Convex	21 (23.6)	27 (30.3)	48 (53.9)	
		Concave	6 (6.7)	8 (9)	14 (15.7)	
		Mixed	10 (11.2)	12 (13.5)	22 (24.7)	
	SP Distal (n (%))	Straight	3 (3.4)	2 (2.2)	5 (5.6)	0.283
		Convex	25 (24.7)	28 (31.4)	53 (59.6)	
		Concave	2 (2.2)	9 (10.1)	11 (12.4)	
		Mixed	8 (9)	12 (13.5)	20 (22.5)	
	Over-contouring (n (%))	0	6 (15.8)	9 (17.3)	15 (16.7)	0.814
		1	3 (8.0)	8 (15.4)	11 (12.2)	
		2	6 (15.8)	9 (17.3)	15 (16.7)	
		3	3 (8.0)	4 (7.7)	7 (7.8)	
		4	20 (52.6)	22 (42.3)	42 (46.7)	
	EARx (degrees) (mean; SD)	Mesial	25.1 (10.2)	28.4 (11.8)	-	0.193
		Distal	23.5 (11.8)	23.8 (12.6)	-	0.961

BoP-: No bleeding on probing group; BoP+: Bleeding on probing group; SD: standard deviation; PPD: Pocket probing depth; BL: Bone level; KM: Keratinized mucosa; SE: Submucosal extension; EA: Emergence angle; SP: Submucosal profile; Over-contouring: Total number of surfaces with over-contouring; HE: External hexagon; IC: Internal connection; EARx: Radiographic emergence angle. † Fisher's exact test. Other statistics are Chi-square.

**Table 2:** Submucosal extension related to bleeding on probing and emergence angle related to bleeding on probing. Number of locations and percentage buccal, palatal, mesial and distal with SE <2 mm or >= 2 mm.

	SE							EA						
	N (%)		BoP-		BoP+		p-value	N (%)		BoP-		BoP+		p-value
	< 2mm	≥ 2mm	< 2mm	≥ 2mm	< 2mm	≥ 2mm		< 30⁰	≥ 30⁰	< 30⁰	≥ 30⁰	< 30⁰	≥ 30⁰	
Buccal	51 (56.7)	39 (43.3)	22 (24.4)	16 (17.8)	29 (32.2)	23 (25.6)	0.841	21 (23.3)	69 (76.7)	7 (7.8)	31 (34.4)	12 (13.3)	40 (44.4)	0.059
Lingual	74 (82.2)	16 (17.8)	34 (37.8)	4 (4.4)	40 (44.4)	12 (13.3)	0.124	43 (47.8)	47 (52.2)	20 (22.2)	18 (20)	23 (25.6)	29 (32.2)	0.431
Mesial	63 (70)	27 (30)	29 (32.2)	9 (10)	34 (37.8)	18 (20.0)	0.264	43 (47.8)	47 (52.2)	20 (22.2)	18 (20)	23 (25.6)	29 (32.2)	0.431
Distal	68 (75.6)	22 (24.4)	32 (35.6)	6 (6.7)	36 (40.0)	16 (17.8)	0.102	55 (61.1)	35 (38.9)	22 (24.4)	16 (17.8)	33 (36.6)	19 (21.1)	0.593

SE: Submucosal extension; Number of locations and percentage buccal, palatal, mesial and distal with EA < 30º or ≥ 30º. EA: Emergence angle; BOP-: No bleeding on probing group; BOP+: bleeding on probing group.

**Table 3:** Correlation between Submucosal extension, Emergence Angle and Emergence Profile and Pink Esthetic Score (PES) and White Esthetic Score (WES).

PES/WES	SE	EA	EPRx
Buccal	0.548	0.032	-
Palatal	0.379	0.613	-
Mesial	0.277	0.677	0.938
Distal	0.715	0.729	0.834

SE: Submucosal extension; EA: Emergence Angle; EPRx: Radiographic Emergence Profile.
